# Implementation of COVID-19 Laboratory Testing Certification Program (CoLTeP) in African Region

**DOI:** 10.3389/fpubh.2022.919668

**Published:** 2022-07-04

**Authors:** Talkmore Maruta, Edwin Shumba, Nqobile Ndlovu, Sikhulile Moyo, Donewell Bangure, Yenew Kebede, Jaurès Arnaud Noumedem Kenfack

**Affiliations:** ^1^Africa Centres for Disease Control and Prevention, Addis Ababa, Ethiopia; ^2^African Society for Laboratory Medicine, Addis Ababa, Ethiopia; ^3^Botswana Harvard AIDS Institute Partnership, Gaborone, Botswana

**Keywords:** COVID-19, laboratory, certification, CoLTeP, non-conformities

## Abstract

**Objectives:**

Coronavirus disease 2019 was declared a global pandemic in March 2020 with correct and early detection of cases using laboratory testing central to the response. Hence, the establishment of quality management systems and monitoring their implementation are critical. This study describes the experience of implementing the COVID-19 Laboratory Testing and Certification Program (CoLTeP) in Africa.

**Methods:**

Private and public laboratories conducting SARS-CoV-2 testing using polymerase chain reaction were enrolled and assessed for quality and safety using the CoLTeP checklists.

**Results:**

A total of 84 laboratories from 7 countries were assessed between April 2021 to December 2021 with 52% of these from the private sector. Among them, 64% attained 5 stars and were certified. Section 4 had the highest average score of 92% and the lowest of 78% in Section 3. Also, 82% of non-conformities (NCs) were related to sample collection, transportation, and risk assessments. Non-availability, inconsistency in performing, recording, instituting corrective actions for failed internal and external quality controls were among major NCs reported.

**Conclusions:**

Laboratories identified for SARS-CoV-2 testing by public and private institutions mostly met the requirements for quality and safe testing as measured by the CoLTeP checklist.

## Introduction

The novel coronavirus disease 2019 (COVID-19) caused by the severe acute respiratory syndrome coronavirus 2 (SARS-CoV-2) was first detected in Africa on the 14 February 2020 in Egypt (1). The coronavirus disease rapidly spread across the globe prompting World Health Organization (WHO) to declare it as a public health emergency of international concern in January 2020 and a global pandemic on 11 March 2020 (1–3). By November 2021, African region had surpassed 8 million cumulative cases and 200,000 deaths (4). In the last 2 years, the coronavirus genome has shown to be highly prone to mutations causing genetic drifts leading to the emergency of variants (5).

The WHO's *strategic preparedness and response plan for COVID-19* highlighted the identification of COVID-19 cases by laboratory testing as central to the pandemic response (6). Testing enables early identification and isolation of cases to slow transmission, the provision of targeted clinical care to those infected, and protection of health systems operations. Inaccurate or clinically unacceptable test results can lead to more dissemination of the virus and serious adverse consequences, including financial loss, trauma, and questions about the integrity of COVID-19 testing programs. It is therefore critical that systems be established that specify requirements for quality, competency, and safety and monitor their implementation by all COVID-19 testing facilities using standard tools.

Under its Saving Lives, Economies, and Livelihoods initiative, Africa Centers for Disease Control and Prevention (Africa CDC) seeks to promote harmonized, standardized, and coordinated entry and exit for travelers in the African Union Member States through digital solutions that harmonize COVID-19 testing results certification (7). The “Trusted Travel, My COVID Pass” tool that it launched simplified verification of public health documentation for travelers during exit and entry across borders (8). The platform digitizes end-to-end laboratory testing, test results certification, and creates a central database of trusted and accredited testing facilities for all member states.

The trusted travel platform requires that there be a database of authorized laboratories certified to conduct COVID-19 testing that port health officials and other stakeholders can use to verify the authenticity of test results. To support this, the African Society for Laboratory Medicine (ASLM) partnered with Africa CDC and launched the COVID-19 Laboratory Testing Certification Program (CoLTeP) in April 2021. The CoLTeP has three (3) main building blocks, its structure, process, and recognition of audited laboratories. Structurally, it is based on recognized international standards for quality and competency of SO 15189, ISO 15190, WHO Biosafety Manual, and the WHO Interim Guidance on SARS-CoV-2 testing. Based on a standard tool, the laboratories are certified as COVID-19 testing laboratories. Process-wise, the coordination of application and enrollment is through a designated office of the Ministry of Health and the deployment of certified auditors for enrolled laboratories by ASLM. The program recognizes and certifies laboratories that attain 5-star rating of the CoLTeP.

This study describes the experience of implementing CoLTeP including lessons learned.

## Materials and Methods

### Data Collection

The enrollment into CoLTeP was open to public and private laboratories conducting SARS-CoV-2 testing using polymerase chain reaction (PCR) based tests.

The CoLTeP assessment checklists was used to assess the laboratory ability to meet the minimum requirements. The data collected from 84 laboratories from eight countries of Botswana, Lesotho Malawi, Mozambique, Niger, Togo, Zambia, and Zimbabwe were analyzed by the elements of the checklists.

### The CoLTeP Assessment Checklist

The CoLTeP assessment checklist was developed based on WHO laboratory assessment tool for laboratories implementing COVID-19 virus testing (9), International Organization for Standardization (ISO) standard 15189:2012 (10), ISO 15190:2020 (11); and the *WHO Laboratory Biosafety Manual*, 4th edition (12). The scored checklist has a total of 56 questions totaling 130 points from 5 sections. At each assessment, the laboratory performance is rated from 0–5-stars using a graduated scale (Table 1). Only laboratories attaining 5-stars are awarded certification as a COVID-19 testing laboratory and enlisted on the Africa Union Trusted Travel Platform.

**Table 1:** COVID-19 testing laboratory testing and certification program score card and the 5 star graduated scale used to audit laboratories in the Africa region, 2021.

**Audit score sheet**
***Section***	***Total Score***
**Section 1: Laboratory Policies and Procedures**	**27**
**Section 2: Laboratory testing capacity**	**29**
**Section 3: Quality Control & Assurance**	**25**
**Section 4: Test result and data management**	**17**
**Section 5: Biosafety and Biosecurity**	**32**
**TOTAL SCORE**	**130**
**No Stars** (0–64 pts) <50%
**1 Star** (65–77 pts) 50–59%
**2 Stars** (78–90 pts) 60–69%
**3 Stars** (91–103 pts) 70–79%
**4 Stars** (104–116 pts) 80–89%
**5 Stars** (117–130 points) ≥90%

*Source: CoLTeP checklist*.

### Enrollment Into CoLTeP

The enrollment was open to public and private laboratories if their applications for enrollment were received by ASLM secretariat from the designated Ministry of Health COVID-19 Testing focal person. Applicant laboratories submitted completed application forms and a certificate of registration/authority to practice as a laboratory for private laboratories for enrollment. The ASLM secretariat communicated with enrolled laboratories on dates of audit and nominated auditors using audit notification letters.

### Training and Deployment of Auditors

The ASLM was responsible for training, certification, and deployment of auditors to applicant laboratories. Auditors nominated by Member States had previous experience in auditing medical laboratory quality management systems. For instance, the ASLM certified Strengthening Laboratory Quality Improvement Process Toward Accreditation (SLIPTA) auditors was considered for training. A 3-day training program was designed by ASLM and deployed to train and certify the auditors who were certified as CoLTeP auditors on completing an online examination administered by the ASLM Academy, a platform where medical laboratory professionals from Africa and across the globe can access online training and information packages that can be used toward continuous professional development (13).

### The CoLTeP Audit Process

After the successful enrollment, ASLM selected and deployed certified auditors. Since the scope of the audit was on the SARS-CoV-2 testing only, one or two auditor(s) were deployed per laboratory for 1 day. At the end of the audit, the auditors compiled audit findings presented them to the audited laboratory before submission to the ASLM secretariat for review, approval, and assignment of appropriate star recognition and possible certification. The feedback on audit findings and laboratory performance was provided at the laboratory and ministry of health levels. On the approval of the report, ASLM would appropriately recognize the laboratory performance as either 0–4-stars or certified as a COVID-19 testing laboratory if attained 5-stars. The laboratories attaining 4-stars were allowed to submit corrective actions virtually within 1 month of the audit and 0–3-star laboratory re-applied for another physical audit at least 3 months from the time of the last audit. The 3 months were to be used for preparation according to audit findings and recommendations.

## Results

The data from 84 laboratories from 8 countries of the African region that were audited between April 2021 to December 2021 is reported. Forty-five (52%) of these laboratories were from the private sector. The average performance as percentage score of the 84 laboratories was 82% with scores ranging from 31 to 100% [mean 82%, standard deviation (SD) 17]. The average score for each of the five checklist sections was above 50% with the highest score in Section 4 (test results and data management) of 91% and lowest in Section 3 Results (quality control and quality assurance), 78% (Table 2).

**Table 2:** The average performance of the 84 laboratories from the African region audited between April–December 2021 in the 5 sections of the checklist, 2021.

**Checklist section score (%)**	**Section 1**	**Section 2**	**Section 3**	**Section 4**	**Section 5**	**Total score**
Mean	80	82	78	91	82	82
Standard deviation	21	14	21	13	16	17
Median	89	86	86	94	88	88
Minimum	19	45	20	47	28	31
Maximum	100	100	100	100	100	100

Over the reported period of implementation of the CoLTeP, none of the laboratories that attained less than 5-stars re-applied for certification.

Most of the laboratories, 54 (64%) attained 5-stars and were awarded certification as a SARS-CV-2 testing laboratory.

The average number of non-conformities per laboratory was 11 (mean 11, SD 10, range 0–40) (Table 3) with 9 (82%) of these being minor NCs Section 1 (Laboratory policies and procedures). Sections Introduction (laboratory policies and procedures) and Section 5 (Biosafety and Biosecurity) Limitations (biosafety and biosecurity) had the highest average number of NCs (Table 2).

**Table 3:** Categorization of different non-conformities identified from the 84 COVID-19 testing laboratories from the African region, 2021.

	**Total_**	**Total**	**Total**	**Total Setion**	**Total Setion**	**Total Setion**	**Total Setion**	**Total Setion**
	**NCs**	**Minor_NCs**	**Major_NCs**	**1 NCs**	**2 NCs**	**3 NCs**	**4 NCs**	**5 NCs**
Mean	11	9	2	3	2	2	1	3
SD	10	9	4	3	2	2	1	2
Minimum	0	0	0	0	0	0	0	0
Maximum	40	40	19	13	9	10	5	13

The reported NCs were categorized into eight areas (Table 4). For the minor NCs under the sample collection and transportation included the absence of sample collection manual and where available, it was not distributed to the sample collection sites and, in some cases, it did not include instructions specific to SARS-CoV-2 sample collection and transportation. Incomplete sample reception records such as the date of samples collection and samples received were also reported. Improper sample packaging and transportation and lack of system for the sample storage and archiving was also identified.

**Table 4:** Summary of reported non-conformities identified from the 84 COVID-19 testing laboratories audited in the African region, 2021.

**Minor NCs**	**Major NCs**
Sample collection and transportation	Sample collection and transportation
Standard operating procedures	Internal and external quality assurance
Biosafety and biosecurity	Biosafety and biosecurity
Reagents and supplies	Reagents and supplies
Personnel management	Personnel management
Internal audits	Internal audits
Corrective actions	Results management

Under biosafety and biosecurity, risk assessments, identification, implementation, and monitoring effectiveness of mitigation measures were not consistently done. Training in biosafety and biosecurity, appointment of biosafety officer, and management of waste were identified as areas of non-conformities. Development, implementation, and tracking corrective actions for their effectiveness was reported. Standard operating procedures were either not available, not communicated to relevant personnel, or outdated. The SARS-CoV-2 reagents and supplies inventory systems for storage and usage tracking was among the minor NCs.

The major NCs included non-availability, inconsistency in performing, recording, and instituting corrective actions for failed Internal and External Quality Controls. Training and competency assessment specific to SARS-CoV-2 testing was a major non-conformity. Conducting, communicating of findings and follow up of internal audits and training of auditors was identified as one of the major NCs. Inconsistent servicing, engineer, and user maintenance for equipment used in SARS-CoV-2 testing was reported from the audits.

## Discussion

Response to COVID-19 pandemic required rapid scale of testing with over a million tests conducted by August 2020 (1). Given the slow uptake of voluntary accreditation of laboratories in the African region, with only 816 laboratories with ISO based accreditation, an alternative to ensure quality of SARS-CoV-2 testing was needed to support the ongoing and rapidly spreading response. Hence, the development and implementation of CoLTeP as a standard framework that offers a rapid evaluation focused on SARS-CoV-2 specific elements that countries could use to authorize testing specifically for SARS-CoV-2. Implementation of a standard approach allowed for the assurance of quality, giving confidence across the territories.

More than 50% of the laboratories evaluated in this period were private, a strategy that most countries adopted to cope with the increased demand for testing which could not be met by the public health facilities. World Health Organization and Africa Centres for Disease Control and Prevention have since advocated for use of faster and easy to use point or near point of care technologies and rapid antigen test platforms to address the long turnaround times that were reported to range from 4.5 to 29 days (14, 15).

At the onset of the pandemic in Africa on the 14 of February 2020, there were only two countries (Senegal and South Africa) able to detect the SARS-CoV-2 virus. However, through the support of WHO and Africa CDC, the capacity rapidly expanded to 38 countries by 20 February 2020 (1). This investment that included the repurposing of existing polymerase chain reaction (PCR) testing laboratories for the human immunodeficiency virus (HIV) is reflected in the majority of audited laboratories (69%) attaining 5-stars and certified. This could also have been influenced by the most audited laboratories being private, and therefore driven by the opportunity for the financial gain, are able to rapidly mobilize resources required to establish quality testing.

The NCs identified in these audited laboratories are consistent with other laboratory systems strengthening initiatives, including the WHO Regional Office for Africa (WHO AFRO) Strengthening Laboratory Quality Improvement Toward Accreditation (SLIPTA) that reported poor performance in areas of internal audits and management of corrective actions. In this study, the additional areas including the sample management are reflective of the lack of knowledge on how to handle the novel virus during the initial phases of the pandemic. The previously unknown SARS-CoV-2 virus presented a biosafety and biosecurity risk that required adjustments in the existing laboratory settings, additional training and equipping testing, sample collectors, and transporters on the requirements for biosafety and biosecurity.

Even though SARS-CoV-2 testing was built on the existing HIV and viral load testing platforms, there was no commercially available internal and external quality control. Although some laboratories developed in-house controls, there were no clear and acceptable methods for their development and storage to meet the requirements of the audits. Hence, this was frequently reported as a major NC. The emergency of SARS-CoV-2 presented a challenge on how results are communicated, with deviations from laboratory to clinician to the laboratory to surveillance teams or directly to the government. As the pandemic progressed, the borders re-opened testing of travels brought another dimension of requesting for testing and communicating results directly to the travelers with no clinician involved. Consequently, laboratories, that had not adjusted their result communication Standard Operating Procedures were found in non-compliance.

The urgent need to initiate testing meant rapid re-purposing of testing staff to cope with increased demand for SARS-CoV-2 testing. The standard procedures for training and competency evaluation were them not followed resulting in non-compliance with standard personal management requirements.

## Limitations

During the period of the study, assessment for certification for SARS-CoV-2 testing was not mandatory; hence, the laboratories that felt they were ready and voluntarily requested for audits, may have created a bias for the high compliance observed.

The authors would also recommend the future studies to explore further challenges associated with implementing systems strengthening program in public health institutions.

## Conclusion

Laboratories identified for SARS-CoV-2 testing by public and private institutions mostly met the requirements for quality and safe testing as measured by the CoLTeP checklist.

## Data Availability Statement

The raw data supporting the conclusions of this article will be made available by the authors, without undue reservation.

## Author Contributions

TM conducted the research and wrote the manuscript. ES conducted the research and reviewed the manuscript. NN, YK, JN, and DB reviewed the manuscript. SM reviewed the manuscript and conducted the statistical analysis. All authors contributed to the article and approved the submitted version.

## Conflict of Interest

TM, DB, YK, and JN are employed in Africa Centres for Disease Control and Prevention. The authors declare that the research was conducted in the absence of any commercial or financial relationships that could be construed as a potential conflict of interest.

## Publisher's Note

All claims expressed in this article are solely those of the authors and do not necessarily represent those of their affiliated organizations, or those of the publisher, the editors and the reviewers. Any product that may be evaluated in this article, or claim that may be made by its manufacturer, is not guaranteed or endorsed by the publisher.
