# Supplementary material for: Implementation of COVID-19 Laboratory Testing Certification Program (CoLTeP) in African Region
Source: Front Public Health. 2022 Jul 4;10:919668. doi: 10.3389/fpubh.2022.919668 (PMC9310066; doi:10.3389/fpubh.2022.919668)
Supplement: Supplementary file 1 [file Data_Sheet_1.PDF]

African Society for Laboratory Medicine

COVID-19 Laboratory Testing Certification Program (CoLTeCP)

COVID-19 LABORATORY TESTING CERTIFICATION ASSESSMENT CHECKLIST

March 2021

LABORATORY/FACILITY PROFILE							
Date of Audit:			Most recent score, star and date		Score	Star	Date
Name(s) and Affiliation(s) of Auditor(s)							
Laboratory/facility Name:						Laboratory/Facility Number	
Laboratory/facility Address:							
Laboratory/Facility Telephone:		Fax:		Email:			
Head of Laboratory/Facility:			Telephone (Head of Laboratory/Facility):			Personal	
						Work	
Laboratory/Facility Level (Tick all that apply)			Type of Laboratory/Laboratory/facility Affiliation (check only one)				
☐ National	☐ Reference	☐ Provincial		☐Public	☐Hospital	☐Private	
☐ District	☐ Zonal	☐ Field		☐ Research	☐Non-hospital Outpatient Clinic	☐Other – Please specify: ______________________ _	

LABORATORY AUDITS

Laboratory audits are an effective means to determine if 1) a laboratory is providing accurate and reliable COVID-19 testing results; 2) a laboratory has an effective biosafety and biosecurity program 3) well-managed and is adhering to good laboratory practices; and 4) identify areas for improvement.

Auditors complete this audit using the methods below to evaluate laboratory operations per checklist items and to document findings in detail.

- Review laboratory documents to verify that they are complete, current, accurate, read and understood by the relevant personnel.
- Review laboratory records: Equipment maintenance records; audit reports, incident reports, personnel files, Internal Quality Control (IQC) records, External Quality Assurance (EQA) records, environmental monitoring records
- Observe laboratory operations to ensure:
    - laboratory testing follows written policies and procedures in pre-analytic, analytic, and post-analytic phases of laboratory testing.
    - laboratory procedures are appropriate for the testing performed.
    - Deficiencies and nonconformities identified are adequately investigated and resolved within the established timeframe.
- Ask open-ended questions to clarify documentation seen and observations made. Ask questions like, “show me how…” or “tell me about…” It is often not necessary to ask all the checklist questions verbatim. An experienced auditor can often learn to answer multiple checklist questions through open-ended questions with the laboratory staff.
- Follow a specimen through the laboratory from collection through registration, preparation, liquating, analysing, result verification, reporting, printing, and post-analytic handling and storing samples to determine the strength of laboratory systems and operations.

- Confirm that each result or batch can be traced back to a corresponding internal quality control (IQC) run and that the IQC was passed. Confirm that IQC results are recorded for all IQC runs and reviewed for validation.
- Confirm Proficieny Testing (PT) results and the results are reviewed and corrective action taken as required.
- Evaluate the quality and efficiency of supporting work areas (e.g., phlebotomy, data registration and reception, messengers, drivers, cleaners, IT,).

AUDIT SCORING

Each item has been awarded a point value of 2, 3 and 5 based upon relative importance and/or complexity. Responses to all questions must be, “yes”, “partial”, or “no”.

- Items marked “*yes*” receive the corresponding point value (2 or 3points). <u>All</u> elements of a question must be present in order to indicate “yes” for a given item and thus award the corresponding points.

<u>**NOTE**</u>: Items that include “tick lists” must receive all “yes” and/or “n/a” responses to be marked “yes” for the overarching item.

- Items marked “*partial*” receive 1 point.
- Items marked “*no*” receive 0 points.

When marking “partial” or “no”, notes should be written in the comments field to explain why the laboratory did not fulfil this item to assist the laboratory with addressing these areas of identified need following the audit.

Where the checklist question does not apply, indicate as NA in the comments section, the star status is then determined using % score

Audit Score Sheet

Section	***Total Points***	***Score***
**Section 1: Laboratory Policies and Procedures**	**27**	
**Section 2: Laboratory testing capacity**	**29**	
**Section 3 : Quality Control & Assurance**	**25**	
**Section 4: Test result and data management**	**17**	
**Section 5: Biosafety and Biosecurity**	**32**	
**TOTAL SCORE**	**130**	

Indicate the laboratory score after scoring all the 5 sections in the table below by highlighting the appropriate star level

**No Stars**<br>(0 – 64 pts)<br>*< 50%*	**1 Star**<br>(65-77 pts)<br>*50 – 59%*	**2 Stars**<br>(78-90pts)<br>*60 – 69%*	**3 Stars**<br>(91-103 pts)<br>*70 – 79%*	**4 Stars**<br>(104-116 pts)<br>*80 – 89%*	**5 Stars**<br>(117-130 points)<br>*≥90%*

6.0 LABORATORY AUDIT

SECTION 1: LABORATORY POLICES AND PROCEDURES					
This section looks at the laboratory documentation and records for COVID-19 testing. The following procedures must be (i) up to date (ii) approved by authorized staff (iii) accessible to relevant staff					
	**Checklist item**	**Yes**	**Partial**	**No**	**Comments**	**Score**
	**Are the following policies and procedures in place:**					
**1.1**	**Is there a current laboratory quality manual, composed of the quality management system's policies and has the manual content been communicated to, understood and implemented by all staff?**					**5**
	The quality manual includes the following elements:	**Yes**	**Partial**	**No**		
	a) Quality policy statement that includes scope of service, standard of service, measurable objectives of the quality management system, and management commitment to compliance.					
	b) Documented policies for the quality management system that meet the requirements of ISO15189:2012 **(Refer to Question 1.5 of this checklist for list of policies required)**					
	c) Description of the quality management system and the structure of its documentation					
	d) Reference to supporting procedures (SOPs), including managerial and technical procedures					
	e) Description of the roles and responsibilities of the laboratory director, or laboratory manager, quality manager, and other key personnel (laboratory to define its key personnel) responsible for ensuring compliance					
	f) Records of review and approval of the quality manual by authorized personnel					
	g) Records to show that the quality manual was communicated to and understood by the lab personnel					
ISO15189:2012 Clause 4.1.2.3 and 4.2.2.2 and 4.3 ***Note:*** *A quality manual must be available that summarizes the laboratory's quality management system, which includes policies that address all areas of the laboratory service, and identifies the goals and objectives of the quality management system. The quality manual must include policies and make reference to processes and procedures for all areas of the laboratory service and must address all the clauses of ISO15189:2012.*						
	Document Control detailing how the laboratory will control all internal and external documents; create documents; identify documents; review documents; approve documents; capture current versions and their distribution by means of a list; handle amendments; identify changes; handle obsolete documents; retain documents prevent the unintended					**2**

	use of any obsolete document and ensure safe disposal of documents?					
ISO15189:2012 Clause 4.3 and 4.13<br>Note: Documents that should be considered for document control are those that may vary based on changes in versions or time. Examples include policy statements, instructions for use, flow charts, procedures, specifications, forms, calibration tables, biological reference intervals and their origins, charts, posters, notices, memoranda, software documentation, drawings, plans, agreements, and documents of external origin such as regulations, standards and text books from which examination procedures are taken.						
1.2	COVID-19 testing procedures written and readily available to staff, as required					**2**
ISO 15189:2012 Clause 5.5.3<br>*Examination procedures shall be documented. They shall be written in a language commonly understood by the staff in the laboratory and be available in appropriate locations*						
1.3	COVID-19 specific sample collection and transportation documented and available to relevant personnel in the laboratory?					**2**
ISO 15189:2012 Clause 5.4.4<br>*The laboratory shall have documented procedures for the proper collection and handling of primary samples. The documented procedures shall be available to those responsible for primary sample collection whether or not the collectors are laboratory staff.*						
1.4	COVID-19 specific sample collection and transportation documented and available to relevant personnel in the collection sites?					**2**
ISO 15189:2012 Clause 5.4.4<br>*The laboratory shall have documented procedures for the proper collection and handling of primary samples. The documented procedures shall be available to those responsible for primary sample collection whether or not the collectors are laboratory staff.*						
1.5	Reporting and communication of COVID-19 testing results?					**2**
ISO 15189:2012 Clause 5.9.1<br>*The laboratory shall establish documented procedures for the release of examination results, including details of who may release results and to whom.*						
1.6	Verification of test procedures for COVID-19 testing used?					**2**
ISO 15189:2012 Clause 5.5.1.2<br>*Validated examination procedures used without modification shall be subject to independent verification by the laboratory before being introduced into routine use.*						
1.7	Biosafety and Biosecurity procedures related to the handling and testing of specimens for COVID-19?					**2**
ISO 15190:2020 Clause 5.2 (e & f)<br>*The plan shall include : procedures for safe practices in handling hazardous materials (f) procedures to prevent theft of high risk and contaminated materials.*						
1.8	Investigating, corrective actions and preventative measures for nonconformities associated with COVID-19 testing results?					**2**
ISO15189:2012 Clause 4.9-11<br>*The laboratory shall have a documented procedure to identify and manage nonconformities in any aspect of the quality management system, including pre-examination, examination or post examination*						
1.9	Safe and secure storage of COVID-19 primary specimens once analysed?					**2**

	Checklist item	Yes	Partial	No	Comments	Score
ISO 15189:2012 Clause 5.4.7 The laboratory shall have procedures and appropriate facilities for securing patient samples and avoiding deterioration, loss or damage during pre-examination activities and during handling, preparation and storage ISO 15189:2012 Clause 5.7.2 The laboratory shall have a documented procedure for identification, collection, retention, indexing, access, storage, maintenance and safe disposal of clinical samples						
1.10	Records control for COVID 19 test results and certificates.					**2**
ISO 15189:2012 clause 4.13 The laboratory shall have a documented procedure for identification, collection, indexing, access, storage, maintenance, amendment and safe disposal of quality and technical records						
1.11	Are the archived COVID-19 documents and records retrievable?					**2**
ISO15189:2012 Clause 4.13 The laboratory shall have a documented procedure for identification, collection, indexing, access, storage, maintenance, amendment and safe disposal of quality and technical records						
	**TOTAL FOR SECTION 1**					**27**
**SECTION 2: LABORATORY TESTING CAPACITY**						
This section looks at the laboratories testing capacity specifically personnel competency, equipment maintenance, infrastructure including the storage facilities and inventory management for COVID-19 testing.						
	**Checklist item**	**Yes**	**Partial**	**No**	**Comments**	**Score**
2.1	Is there space allocated for COVID-19 testing?					**2**
ISO 15190:2020 Clause 12: Laboratory activity workspace and equipment e.g chairs , laboratory workstations , computer keyboards and displays) as well as vibration producing and ultra sound equipment e.t.c. shall be designed or positioned to reduce risks of ergonomic distress disorders and accidents						
2.2	If the laboratory uses PCR tests, is workflow arrangement allow flow of work in a uni-direction and areas segregated appropriately.					**2**
ISO 15189:2012 clause 5.2.6 There shall be effective separation between laboratory sections in which there are incompatible activities. Procedures shall be in place to prevent cross-contamination where examination procedures pose a hazard or where work could be affected or influenced by not being separated						
2.3	Are personnel trained in performing COVID-19 virus testing with documented up to date records?					**3**
ISO 15189:2012 Clause 5.1.6 Following appropriate training, the laboratory shall assess the competence of each person to perform assigned managerial or technical tasks according to established criteria.						
		**Yes**	**Partial**	**No**		
2.4	Are records of personnel conducting COVID-19 testing maintained and do they include the following?					**3**
	a) Educational and professional qualifications?					
	b) Copy of certification or license to practice, when applicable?					
	c) Previous work experience e.g. CV?					

	d) Biosafety and Biosecurity training records					
ISO15189:2012 Clause 5.1.9<br>*Records of the relevant educational and professional qualifications, training and experience, and assessments of competence of all personnel shall be maintained.*						
2.5	Is competence done and records maintained for COVID-19 testing personnel.					**2**
ISO 15189:2012 Clause 5.1.6<br>*Following appropriate training, the laboratory shall assess the competence of each person to perform assigned managerial or technical tasks according to established criteria.*						
2.6	Is a functional and validated biological safety cabinet (BSC) Class II available?					**2**
ISO 15190:2020 Clause 7.7 (c)<br>*Biological safety cabinets shall be tested and certified upon installation, when moved or repaired and annually ; records shall be kept of the inspection and any functionality testing results*						
2.7	Is there a a preventive maintenance schedule in place for COVID-19 equipment and implemented following manufacturer recommendations					**2**
ISO15189:2012 Clause 5.3.1.5<br>*The laboratory shall have a documented programme of preventive maintenance which, at a minimum, follows the manufacturer's instructions*						
2.8	Is critical equipment used for COVID-19 testing supported by uninterrupted power source (UPS) systems?					**2**
ISO15189:2012 Clause 4.1.1.4(a);(n); 4.1.2.1(i);<br>*Note: Interruption to services is considered when a laboratory cannot release results to their users. Testing services should not be subject to interruption due to equipment mal functions. Contingency plans must be in place, in the event of equipment failure, for the completion of testing. In the event of a testing disruption, planning may include the use of a back-up instrument, the use of a different testing method, the referral of samples to another laboratory*						
2.9	Is there allocated space for storage of COVID-19 testing reagents and consumables?					**2**
ISO15189:2012 Clause 5.3.2.2<br>*Where the laboratory is not the receiving facility, it shall verify that the receiving location has adequate storage and handling capabilities to maintain purchased items in a manner that prevents damage or deterioration*						
2.10	Are all reagents/test kits for COVID-19 testing in use (and in stock) currently within the manufacturer-assigned expiration or within stability?					**2**
ISO15189:2012 Clause 5.3.2.3<br>*Each new formulation of examination kits with changes in reagents or procedure, or a new lot or shipment, shall be verified for performance before use in examinations. Consumables that can affect the quality of examinations shall be verified for performance before use in examinations.*						
2.11	Are expired COVID-19 products labelled and disposed properly?					**2**
ISO15189:2012 Clause 5.3.2.7<br>***Note**: Expired products should be disposed of properly and records maintained. If safe disposal is not available at the laboratory, the manufacturer/supplier should take back the expired stock at the time of their next delivery.*						
2.12	Does the laboratory maintain inventory records for each COVID-19 reagent and consumable that contributes to the performance of examinations?					**2**
ISO15189:2012 Clause 4.13; 5.3.2.7; 5.3.2.4<br>***Note:** All incoming orders should be inspected for condition and completeness of the original requests, receipted and documented*						

appropriately; the date received in the laboratory and the expiry date for the product should be clearly indicated.						
2.13	Are internal audits conducted to evaluate continued compliance of the COVID-19 testing processes to the laboratory quality management system?	**Yes**	**Partial**	**No**		**3**
	a) An audit schedule documented and followed					
	b) Audit findings documented and communicated to relevant staff					
	c) Identified non-conformities investigated and corrective actions implemented					
	d) Corrective actions implemented monitored for their effectiveness					
ISO15189:2012 Clause 4.14.6<br>*The Laboratory shall assess all steps in for all its processes (pre-analytical, analytical and post analytical) for areas of potential pitfalls e.g. pre-analytical step of sample collection, potential pitfalls could be; wrong sample collected, sample collected in wrong container, sample collected at wrong time. Post analytical could be; result sent to wrong patient, results sent outside of TAT. The Lab must assess all steps, list potential pitfalls and document action taken to prevent these from occurring.* ***Note: Risks should be graded and acted upon as per their grading***						
	**TOTAL FOR SECTION 2**					**29**
**SECTION 3: QUALITY CONTROL AND QUALITY ASSURANCE**						
This section looks at the COVID-19 quality control and quality assurance practices including the use of internal quality control, use of proficiency testing samples and the investigation and resolution of quality issues.						
	**Checked item**	**Yes**	**Partial**	**No**	**Comments**	**Score**
**3.1**	**Are all COVID-19 testing methods validated/verified and is documented evidence available?**					**3**
	Are specific verification/validation protocols in place for all COVID-19 testing methods?					
	Is validation/verification performed for all COVID-19 testing methods with documented validation/verification reports available					
	Are validation/verification reports signed and approved by authorized personnel?					
ISO15189:2012 Clause 5.3.1.2; 5.5.1<br>*Note: Newly introduced methods or equipment must be verified onsite to ensure that their introduction yields performance equal to o r better than the previous method or equipment. Manufacturers' validation may be used. Back up equipment must also be included in verification procedures.*						
3.2	Are all samples received captured appropriately with records of date collected, date and time received, person receiving					**2**
ISO 15189:2012 Clause 5.4.6 (d)<br>*All samples received are recorded in an accession book, worksheet, computer or other comparable system. The date and time of receipt and/or registration of samples shall be recorded. Whenever possible, the identity of the person receiving the sample shall also be recorded.*						

3.3	Is an acceptance/rejection criterion applied at the time of receiving samples?					**2**
ISO 15189:2012 Clause 5.4.6 (e)<br>*Authorized personnel shall evaluate received samples to ensure that they meet the acceptance criteria relevant for the requested examination(s)*						
3.4	Are COVID-19 samples tested within recommended time from collection?					**2**
ISO15189 clause 5.4.5(a &b)<br>*Tthe laboratory shall ensure that samples are tested a) within a time frame appropriate to the nature of the requested examinations and the laboratory discipline concerned; b) within a temperature interval specified for sample collection and handling and with the designated preservatives to ensure the integrity of samples;*						
3.5	Is there monitoring of environmental conditions where COVID-19 testing is performed, and reagents and consumables are stored.					**2**
ISO15189:2012 clause 5.2.6<br>*The laboratory shall monitor, control and record environmental conditions, as required by relevant specifications or where they may influence the quality of the sample, results, and/or the health of staff*						
3.6	Are internal quality controls (IQC) material specimens included when performing COVID-19 testing?					**3**
ISO15189:2012 clause 5.6.2.2<br>*The laboratory shall use quality control materials that react to the examining system in a manner as close as possible to patient samples.*						
3.7	Are failed IQC investigated, corrective and preventative actions implemented					**3**
ISO 15189:2012 clause 5.6.2.3<br>*The laboratory shall have a procedure to prevent the release of patient results in the event of quality control failure. When the quality control rules are violated and indicate that examination results are likely to contain clinically significant errors, the results shall be rejected and relevant patient samples re-examined after the error condition has been corrected and within-specification performance is verified. The laboratory shall also evaluate the results from patient samples that were examined after the last successful quality control event.*						
3.8	Does the laboratory participate in EQA program for all of its COVID-19 test methods?					**3**
ISO 15189:2012 clause 5.6.3.1<br>*The laboratory shall participate in an interlaboratory comparison programme(s) (such as an external quality assessment programme or proficiency testing programme) appropriate to the examination and interpretations of examination results. The laboratory shall monitor the results of the interlaboratory comparison programme(s) and participate in the implementation of corrective actions when predetermined performance criteria are not fulfilled*						
3.9	Are failed EQA investigated, corrective and preventative actions implemented					**3**
ISO 15189:2012 clause 5.6.3.1<br>*The laboratory shall participate in an interlaboratory comparison programme(s) (such as an external quality assessment programme or proficiency testing programme) appropriate to the examination and interpretations of examination results. The laboratory shall monitor the results of the interlaboratory comparison programme(s) and participate in the implementation of corrective actions when predetermined performance criteria are not fulfilled*						
3.10	Are corrective actions implemented for the identified non-conformities monitored for their effectiveness.					**2**
ISO15189:2012 Clause 5.6.3<br>*Note:The laboratory should handle, analyze, review and report results for proficiency testing in a manner similar to regular patient testing. Investigation and correction of problems identified by unacceptable proficiency testing should be documented. Acceptable results showing bias or trends suggest that a problem should also be investigated.*						

	**TOTAL FOR SECTION 3**					**25**
	**SECTION 4: TEST RESULTS AND DATA MANAGEMENT**					
This section looks at COVID-19 information management in the laboratory.						
	**Checked item**	**Yes**	**Partial**	**No**	**Comments**	**Score**
4.1	Are all original observations/results of the laboratory recorded legibly?					**2**
ISO 15189:2012 clause 5.9.1 (c)<br>*Results are legible, without mistakes in transcription, and reported to persons authorized to receive and use the information*						
4.2	Are the results reviewed and approved by authorized personnel before being released?					**2**
ISO 15189:2012 clause 5.7.1<br>*The laboratory shall have procedures to ensure that authorized personnel review the results of examinations before release and evaluate them against internal quality control and, as appropriate, available clinical information and previous examination*						
4.3	Is there a system for immediate notification of relevant authorities for all confirmed positive COVID-19 test results?					**2**
ISO 15189:2012 clause 5.9.1<br>*The laboratory shall establish documented procedures for the release of examination results, including details of who may release results and to whom.*						
4.4	Does the laboratory submit COVID -19 routine testing data to the National Surveillance system?					**3**
4.5	Are test results released with stated turnaround times					**2**
ISO15189:2012 Clause 4.14.7<br>*The laboratory, in consultation with the users, shall establish turnaround times for each of its examinations that reflect clinical needs. The laboratory shall periodically evaluate whether or not it is meeting the established turnaround times.*						
4.6	Are access and modification of patient data protected (for paper-based and/or electronic system)?					**2**
ISO 15189:2012 Clause 5.10.2<br>*The laboratory shall ensure that the authorities and responsibilities for the management of the information system are defined, including the maintenance and modification to the information system(s) that may affect patient care*						
4.7	Is efficient back-up in place to prevent loss of patient result data in case of theft or other incident (for paper-based and/or electronic system)					**2**
ISO 15189:2012 Clause 5.10.3<br>*The laboratory shall have documented contingency plans to maintain services in the event of failure or downtime in information systems that affects the laboratory's ability to provide service.*						
4.8	Are records kept of all COVID-19 positive samples (date stored person storing, date and who retrieved, date and who disposed)					**2**
ISO 15189:2012 clause 4.13<br>*The laboratory shall have a documented procedure for identification, collection, indexing, access, storage, maintenance, amendment and safe disposal of quality and technical records*						

		Yes	Partial	No	Comments	Score
	**TOTAL FOR SECTION 4**					**17**
**SECTION 5: BIOSAFETY AND BIOSECURITY**						
This section looks at the laboratories testing capacity specifically personnel competency, equipment maintenance, infrastructure including the storage facilities and inventory management						
	**Checked item**	**Yes**	**Partial**	**No**	**Comments**	**Score**
5.1	Is there a designated and trained biosafety and biosecurity officer?					**3**
ISO 15190:2020 Clause 5.5 An appropriately qualified and experienced laboratory safety officer shall be designated to assist the laboratory management with safety issues and report directly to the laboratory management						
5.2	Has a risk assessment related to the procedures being undertaken for COVID-19 testing in the laboratory covering preanalytical, analytical and post analytical been performed, documented and mitigation actions implemented					**3**
ISO 15190:2020 Clause 6.3 Risk assessments shall be required for every examination procedure or service or group of related examinations or services with the aim that exposure to hazards be eliminated wherever possible. Risk assessment require evaluation of both task –specific and environmental hazards						
5.3	Are mitigations measures implemented monitored for their effectiveness?					**2**
ISO15190:2020 clause 6.4 There shall be careful monitoring of action plans implementation and the program should be of constant improvement in the risk reduction process.						
5.4	Are samples received in the laboratory appropriately package following triple packaging?					**2**
ISO15190:2020 Clause 16: All samples shall be transported to the laboratory in such a manner as to prevent contamination of workers , patients or environment						
5.5	Is personnel collecting and transporting samples for COVID-19 received appropriate training in biosafety and biosecurity?					**2**
ISO 15190:Clause 5.9.2 (e) A comprehensive safety training program shall address at a minimum fire prevention and preparedness , chemical and radiation safety, biological hazards, infection prevention, occupational health and safety (e.g vaccines) first aid and environmental protection						
5.6	Are work surfaces decontaminated with appropriate disinfectants?					**2**
ISO 15190:2020 clause 7.5 The organization shall establish and maintain validated procedures to ensure that appropriate methods for decontamination and inactivation are chosen and implemented effectively						
5.7	Does the laboratory staff use PPE appropriately while working in the laboratory?					**2**
ISO15190:2020 Clause 7.6 Standard precaution, routine practices and additional precautions shall be used with all patients, all patient samples at all times. These precautions shall include airborne, droplet and contact precautions. In order to prevent exposure to blood , body fluid , secretions and excretions personal protective equipment (PPE) shall me implemented						

5.8	Do staff adhere to biosafety and biosecurity procedures as stated by the laboratory?					**2**
ISO15190:2020 Clause 7.6<br>*Standard precautions, routine practices and additional precautions shall be used with all patients, all patient samples at all times. These precautions shall include airborne, droplet and contact precautions. All samples , cultures and waste shall be assumed to contain viable biological agents that can be associated with transmission of infectious disease and shall be handled in a safe manner*						
5.9	Are appropriate PPE for the handling and testing of specimens for COVID-19 virus available?					**2**
ISO15190:2020 Clause 15.2<br>*The laboratory shall ensure that ample supply of clean PPE appropriate to the level of risk is available for those working or visiting the laboratory*						
5.10	Is biohazard waste generated in the laboratory handled properly					**3**
	Appropriate waste containers available in areas where waste is generated					
	Waste appropriately segregated at the point it is generated					
	Waste properly and securely stored before disposal					
	Waste properly treated before disposal					
	Waste collection for disposal documented (time and person collecting)					
ISO 15190:2020 Clause 17:<br>*It is presupposed that laboratory waste disposal policies and procedures are developed with an awareness of the applicable statutory requirements*						
5.11	Are access control measures in place and implemented?					**2**
ISO 15190:2020 Clause 4.3.3<br>*: Laboratory access shall be restricted to authorized personnel*						
5.12	Is access to COVID-19 positive samples storage areas controlled?					**2**
ISO 15190:2020 Clause 4.3.3:<br>*Additional security measures such as lockable doors ,freezers , limited access to specific personnel etc can be required when handling and storing highly hazardous samples ,cultures , chemical reagents or supplies as indicated by the risk assessment*						
5.13	Are accidents/incident related to biosafety and biorisk managed appropriately (i.e. reported, recorded, investigated, and leading to preventive or corrective actions)?					**3**
ISO15190:2020 Clause 19:<br>*the laboratory shall have a programme for reporting laboratory incidents ,accidents, near misses and occupational illness as well as potential hazard with responsibilities of laboratory staff, laboratory safety officer and laboratory management clearly specified.*						
5.14	Does the laboratory have access to high temperature incinerator?					**2**
ISO15190:2020 Clause17.3<br>*General precautionary waste disposal measures for hazardous waste shall be implemented.*						
	**TOTAL FOR SECTION 5**					**32**
	**GRAND TOTAL**					
