# Supplementary material for: Implementation of COVID-19 Laboratory Testing Certification Program (CoLTeP) in African Region
Source: Front Public Health. 2022 Jul 4;10:919668. doi: 10.3389/fpubh.2022.919668 (PMC9310066; doi:10.3389/fpubh.2022.919668)
Supplement: Supplementary file 2 [file Data_Sheet_2.PDF]

COVID-19 Laboratory Testing Certification Program (CoLTeCP)

Virtual Auditor Training Program

May 2021

1.0 BACKGROUND

The African Society for Laboratory Medicine (ASLM) is a pan-African professional body with a mission to advance professional laboratory medicine practice, science, systems and networks in Africa needed to support preventive medicine, quality care of patients and disease control through partnership with government and relevant organizations (www.aslm.org).

Taking into account the health challenges faced by the African continent and the necessity for an accountability framework for health security to protect citizens of the continent, African Union (AU) Heads of States and Governments approved the establishment of the Africa Centres for Disease Control and Prevention (Africa CDC) through the **XXVI Assembly/AU/DEC.589** which adopted the Statute of the Africa CDC and itsframework of operations. The Africa CDC was officially launched on the 31st of January 2017. Article 3 of the Statute gives the Africa CDC mandate to promote the prevention and control of diseases as well as promote partnership and collaboration among member states to address the emerging and endemic diseases and public health emergencies.

The WHO Strategic Preparedness and Response Plan for COVI9-19[1] highlighted identification of COVID-19 cases by laboratory testing as central to the pandemic response. Testing enables early identification andisolation of cases to slow transmission, the provision of targeted clinical care to those infected, and protection ofhealth systems operations. Inaccurate or clinically unacceptable test results can lead to serious adverse consequences, including financial loss, trauma and questions about the integrity of COVID-19 testing programs.It is therefore critical that systems be established that specify requirements for quality, competency and safety and monitors their implementation by all COVID-19 testing facilities using standard tools.

Under its Saving Lives, Economies and Livelihoods initiative[2], Africa Centres for Disease Control and Prevention (Africa CDC) seek to promote harmonized, standardized and coordinated entry and exit for travellers in African Union Member States through digital solutions. Among its objectives is to harmonize COVID-19 testing certification. In collaboration with partners PanaBIOS and Econet Wireless, African Union (AU) and Africa CDC launched the *"Trusted Travel, My COVID Pass*"[3] tool to simplify verification of public health documentation for travellers during exit and entry across borders. The platform digitizes end-to-end laboratory testing, test results certification and creates a central database of trusted and accredited testing facilities for all Member States.

---

[1] World Health Organization. Laboratory testing strategy recommendations for COVID-19. Interim guidance. https://apps.who.int/iris/bitstream/handle/10665/331509/WHO-COVID-19-lab_testing-2020.1-eng.pdf?sequence=1&isAllowed=y
[2] Africa CDC. Saving lives, Economies and Livelihoods. https://africacdc.org/download/saving-lives-economies-and-livelihoods-in-africa/
[3] Africa CDC. Trusted Travel Platform. https://africacdc.org/trusted-travel/

The Trusted Travel Platform requires that there be a database of authorised laboratories certified to conduct COVID-19 testing that port health officials and other stakeholders can use to verify authenticity of test results. To support this, ASLM will implement the COVID-19 Laboratory Testing Certification Program (CoLTeCP). ALSM in collaboration with Africa CDC, will host a workshop on Training of Laboratory Assessors for the COVID-19 Laboratories Testing Certification Program for the Eastern Africa Region on 31st May-2nd June 2021 virtually.

2.0 TRAINING AND CERTIFICATION OF AUDITORS

A standard checklist was developed to evaluate laboratories that have been prioritized by the Ministry of Health for COVID-19 testing. The checklist evaluates laboratories in five critical areas of policies and procedures, laboratory testing capacity, quality assurance and quality control, data management and biosafety and biosecurity. The scored checklist recognizes the incremental implementation of COVID-19 testing requirementsas stipulated by WHO in their interim guidance as well as ISO 15189, ISO 17025, ISO 15190, WHO Biosafety Manual, 4th Edition and Good Laboratory Practices. Only facilities achieving 90% are certified as COVID-19 testing facilities and listed on the Africa CDC Trusted Platform. To ensure sustainability as well rapid scalability, ASLM will use locally trained and certified assessors to conduct assessments.

3.0 OBJECTIVES OF THE TRAINING

The 3-day Virtual training program will seek to familiarize auditors with the

1. ASLM Covid-19 Laboratory Testing Program (CoLTeP)
2. Covid-19 Laboratory Testing requirements
3. CoLTeP Checklist
4. CoLTeP Audit Process

4.0 TARGET PARTICIPANTS

The workshop will engage about 24 participants from 10 Eastern Africa Member States (Kenya, Tanzania, Uganda , Rwanda, Burundi, Ethiopia, Eretria, Somalia , South Sudan and Djibouti) and technical staff from Africa CDC and ASLM secretariat.

4.2 SELECTION CRITERIA FOR AUDITORS

Candidates will be selected based upon the principle of ensuring institutionalization of the program within Ministry of Health as well as build upon the already existing capacity from the World Health Organization Regional Office for Africa (WHO/AFRO) Strengthening Laboratory Quality Improvement Process Towards Accreditation (SLIPTA) program,. ASLM Certified SLIPTA auditors will be prioritized for training as they already have the requisite knowledge of international standard for quality as well as audit skills.

The following criteria will be applied for selection of trainee auditors

1. Nominated by Ministry of Health
2. Certified ASLM SLIPTA Auditor

5.0 TRAINING CONTENT

The virtual training will be based on requirements for COVID-19 testing as stipulated in the WHO Interim Guidance for COVI-19 Testing, ISO 15189, ISO 17025 and ISO 15190 standards and the WHO Biosafety Manual, 4th Edition. See Annex 1: Training agenda

6.0 LOGISTICS

- Venue: Virtual Training. Connection link to be shared with selected participants
- Working Language: English

7.0 ANNEXES

7.1 Annex 1: Training Agenda

**Regional CoLTeP ToT Program**			
**Monday**		**Duration**	**Facilitator**
8:30 am	Introductions	0:15	All
8:45 am	Official Opening Remarks	0:15	Africa CDC, ASLM
9:00 am	Workshop Overview	0:15	Facilitator
9:15 am	Overview of the CoLTeP Program	0:45	Facilitator
10:00 am	WHO COVI-19 Testing Interim Guidalines	1:30	Facilitator
**11:30AM**	**Health Break / Group Photo**	0:10	
11:40 am	Overview of WHO Biosafety Manual 4th Edition	1:50	Facilitator
1:30 pm	Adjourn		
**Tuesday**		**Duration**	**Facilitator**
8:30 am	Review of Day 1	0:15	
8:45 am	Review of the CoLTeP checklist	2:45	
**11:30AM**	**Health Break**	**0:10**	
11:40 am	Review of the CoLTeP checklist	1:50	Facilitator
1:30 pm	Adjourn		All
**Wednesday**		**Duration**	**Facilitator**
8:30 am	Review of Day 2	0:15	All
8:45 am	Review of the CoLTeP Process	2:15	Facilitator
**11:00 am**	**Health Break**	**0:15**	
11:15 am	Final Exam	1:00	All
12:15 pm	**Closing Remarks & Adjourn**	1:00	Africa CDC, ASLM
